# Supporting caregivers of veterans with Alzheimer’s disease and traumatic brain injury: study protocol for a randomized controlled trial

**DOI:** 10.1186/s13063-020-4199-1

**Published:** 2020-04-19

**Authors:** Jennifer L. Carnahan, Katherine S. Judge, Joanne K. Daggy, James E. Slaven, Nicki Coleman, Emily L. Fortier, Christopher Suelzer, Nicole R. Fowler

**Affiliations:** 1 Department of Medicine, Indiana University School of Medicine, 1101 West 10th Street, Indianapolis, IN 46202 USA; 2 Division of General Internal Medicine and Geriatrics, Indianapolis, USA; 3 Regenstrief Institute, Indiana University Center for Aging Research, Indianapolis, USA; 4 Department of Psychology, College of Sciences and Health Professions, Cleveland State University, 1836 Euclid Avenue, Cleveland, OH USA; 5 Department of Biostatistics, Indiana University School of Medicine, 410 West 10th Street, Suite 3000, Indianapolis, IN 46202 USA; 6 Richard L. Roudebush VA Medical Center Research Services, 1481 West 10th Street, Indianapolis, IN 46202 USA

**Keywords:** Alzheimer’s disease, Dementia, Traumatic brain injury, Quality of life, Veterans, Caregivers, Collaborative care

## Abstract

**Background:**

Patients with Alzheimer’s disease and related dementias (ADRD) and traumatic brain injury (TBI) and their caregivers require cognitive and behavioral symptom management, interdisciplinary care, support for caregivers, and seamless care coordination between providers. Caring for someone with ADRD or TBI is associated with higher rates of psychological morbidity and burden, social isolation, financial hardship, and deterioration of physical health. Tremendous need exists for primary care–based interventions that concurrently address the care needs of dyads and aim to improve care and outcomes for both individuals with ADRD and TBI and their family caregivers.

**Methods:**

The Aging Brain Care Acquiring New Skills While Enhancing Remaining Strengths (ABC ANSWERS) study is a randomized controlled trial that tests the effectiveness of an intervention based on two evidence-based programs that have been developed for and previously tested in populations with ADRD, TBI, stroke, and late-life depression and/or who have survived an intensive care unit stay. This study includes 200 dyads comprised of a veteran with a diagnosis of ADRD or TBI and the veteran’s primary informal caregiver. Dyads are randomized to receive the ABC ANSWERS intervention or routine Veterans Health Administration (VHA) primary care with a standardized educational and resource information packet. Data collection occurs at baseline and three follow-up time points (3 months, 6 months, and 12 months). The primary outcome is caregiver quality of life (QoL). A secondary measure for the caregiver is caregiver burden. Secondary measures for both the veteran and caregiver include symptoms of depression and anxiety.

**Discussion:**

The ABC ANSWERS intervention integrates common features of an evidence-based collaborative care model for brain health while concurrently attending to the implementation barriers of delivering care and skills to dyads. We hypothesize that caregivers in dyads randomized to the ABC ANSWERS program will experience higher levels of QoL and lower levels of depression, anxiety, dyadic strain, and caregiver burden at 12 months than those receiving usual VHA primary care.

**Trial registration:**

ClinicalTrials.gov, NCT03397667. Registered on 12 January 2018.

## Background

The number of Americans aged 65 years or older with Alzheimer’s disease and related dementias (ADRD) is expected to grow to more than 13.8 million by 2050 [1]. Although the prevalence of ADRD in veterans is currently similar to that in the overall population, it is anticipated to grow dramatically as the population ages and as a result of the increased incidence of traumatic brain injury (TBI), a known risk factor for developing ADRD [2, 3]. Of the more than 2.3 million soldiers who have been deployed to Iraq and/or Afghanistan [1], 379,519 have been diagnosed with TBI since 2000 [4]. Individuals who experience multiple concussions or sustain a single moderate to severe-TBI have a two- to fourfold increased risk of developing subsequent neurodegenerative diseases [5]. Furthermore, a history of TBI within 10 years of an ADRD diagnosis is associated with more rapid functional impairment progression [6].

Individuals with ADRD and TBI experience increasing cognitive and functional limitations that may induce symptoms of depression and anxiety. A recent systemic review and meta-analysis reported an overall prevalence of 49% for apathy in patients with ADRD, whereas 42% had depression, 40% had aggression, 39% had anxiety, and 39% had sleep disorders [7]. Similarly, patients with TBI frequently experience agitation (11–70%), aggression (25–39%), irritability (29–71%), alcohol abuse (7–26%), drug abuse (2–20%), apathy (20–71%), depression (12–76%), anxiety (0.8–24.5%), post-traumatic stress (11–18%), and obsessive-compulsive disorder (1.2–30%) [8]. Thus, interventions aimed at cognitive symptoms that are also targeted at improving quality of life (QoL) of patients with ADRD and patients with TBI are urgently needed.

Concurrent with the increasing number of patients with ADRD and/or TBI, more than 16 million civilians in the United States provide unpaid care for someone with ADRD, accounting for 83% of the care these patients receive [9]. There also are approximately 5.5 million U.S. military caregivers of veterans, of whom 1.1 million are caring for post-9/11 veterans [10]. Growing evidence indicates that family caregivers of patients with ADRD and TBI provide more assistance with activities of daily living (ADL) and spend more time on the management of the patients’ safety and behavioral symptoms than caregivers of people without cognitive impairments [11, 12]. Additionally, caregivers face daunting tasks of ensuring access to care by identifying and coordinating supportive services, facilitating health care visits, advocating for veterans, and serving as a proxy for health care and financial decisions. Thus, collaborative partnerships between health care professionals, specifically in primary care, and informal caregivers are crucial for the comprehensive management of these veterans [12].

The burden and stress experienced by ADRD and TBI caregivers are often higher than for caregivers of individuals with physical disabilities [13]. Specifically, caregivers of individuals with brain disorders experience poorer health outcomes, both physical and emotional, resulting in higher health care use than for other caregivers. They also spend significantly more hours per week providing care [13]. As a result, they experience excessive emotional burden and are at higher risk of depression than other caregivers. ADRD and TBI caregivers also report significantly greater employment complications, including having to retire early, turning down a promotion, losing benefits, and in turn financial hardship [13].

Early intervention can improve the QoL and functional outcomes of patients with ADRD and TBI, resulting in reduced health care costs [14, 15]. Most interventions targeting patients with ADRD or patients with TBI have focused on cognitive rehabilitation techniques to improve learning, memory, and performance of ADL/instrumental ADL, such as psychoeducational and psychotherapeutic approaches and integrated behavioral health programs [16]. Although research supports these interventions, few have been validated through randomized controlled trials.

Given the significant overlap in needs of ADRD and TBI caregivers, many caregiver interventions have been tested in both populations. Interventions focused on caregiver education have been shown to improve caregiver confidence [17], delay time to nursing home placement [18], and improve caregiver health [19]. Additionally, interventions that go beyond education to include behavioral management, cognitive training, and collaborative care management have demonstrated reduced caregiver physical morbidity [20], depression and depressive symptoms [10, 21], and anger and fatigue [22]. Interventions aimed at the physical health and well-being of caregivers have revealed that support and activity interventions improve caregivers’ physical health [23] as well as QoL and overall subjective well-being [10]. Further interventions aimed at caregivers’ well-being also have demonstrated that the experience of caregiving can add meaning to their lives through opportunities to deliver something important to a family member they love [20, 24].

Although the efficacy of interventions for caregivers has been well studied and holds promise to reduce caregiver burden, few studies have taken the next steps in testing the optimal strategies of scaling evidence-based interventions and embedding them into existing Veterans Health Administration (VHA) primary care. To fill the translational gap between clinical care in the research setting and real community practice, our research team is conducting a randomized controlled trial, the Aging Brain Care Acquiring New Skills While Enhancing Remaining Strengths (ABC ANSWERS) study, to examine the impact of integrating two evidence-based programs: a macro, systems-based collaborative care intervention (ABC Program) and a micro, strength-based intervention (ANSWERS) [2, 4, 10, 25–32] into one program delivered within primary care. The primary outcome is caregivers’ QoL. Secondary caregiver outcomes include symptoms of depression and anxiety, dyadic strain, and caregiver burden. Secondary veteran outcomes are symptoms of depression and anxiety. We hypothesize that caregivers in dyads randomized to the ABC ANSWERS program will experience higher levels of QoL and lower levels of depressive and anxiety symptoms, dyadic strain, and caregiver burden at 12 months than those receiving usual VHA primary care. Additionally, we hypothesize that veterans randomized to the ABC ANSWERS program will experience higher levels of QoL and lower levels of depressive and anxiety symptoms at 12 months than those receiving usual VHA primary care.

## Methods

### ABC ANSWERS study design

**Fig. 1:**
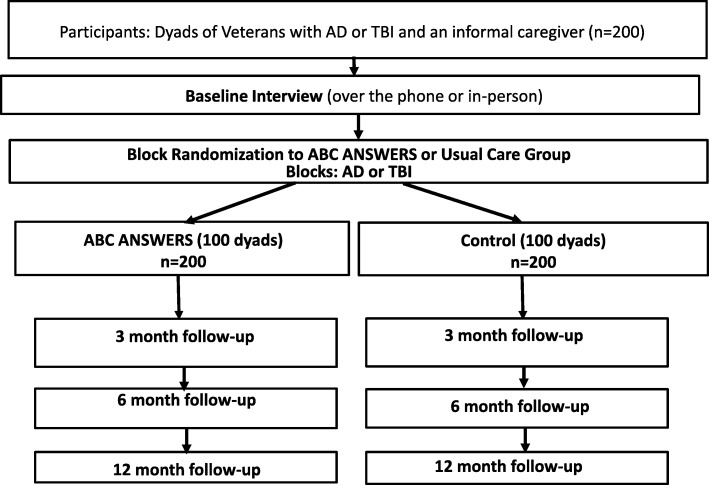
Aging Brain Care Acquiring New Skills While Enhancing Remaining Strengths study design

ABC ANSWERS is a 3-year randomized controlled trial that includes 200 dyads, comprised of a veteran with a diagnosis of ADRD or TBI and the veteran’s primary informal caregiver. Within each stratum defined by the veteran’s primary diagnosis (ADRD or TBI), dyads are randomized 1:1 to receive ABC ANSWERS or usual primary care provided by VHA Patient Aligned Care Teams (PACTs). Each dyad is followed for 12 months. An overview of the study design is depicted in Fig. 1. This study protocol has followed the Standard Protocol Items: Recommendations for Interventional Trials (SPIRIT) statement guidelines (Additional file 1). The trial will be conducted and reported according to the reporting of pragmatic trials in an extension of the Consolidated Standards of Reporting Trials (CONSORT) statement. The study has been approved by the institutional review board (IRB) of Indiana University (IRB 1706902669R001); the Indianapolis Veteran Affairs Medical Center VA Research Subcommittee; and the U.S. Army Medical Research and Material Command, Office of Research Protections, Human Research Protection Office (HRPO AZ160032).

### Setting and study population

The ABC ANSWERS trial is being conducted in primary care clinics affiliated with a level 1 complexity VA medical center in the Midwest. Recruitment for ABC ANSWERS occurs through VHA primary care PACTs and PACTs at affiliated community-based outpatient clinics, in which about 60% of the population are veterans living in rural areas. We also recruit from the PACTs at the extender primary clinic.

### Eligibility

#### Inclusion criteria

Veterans are included if they (1) have an ADRD or TBI diagnosis at any stage or level of impairment of their illness, (2) receive primary care services from VHA, (3) can identify a study partner who is a family or friend caregiver, (4) do not reside in a nursing home, (5) speak and read English, and (6) have access to a telephone. Caregivers are included if they (1) have been identified as the primary person who provides, or would provide if needed, the majority of assistance to the individual; (2) have plans of providing care at least for 1 year or longer; (3) are available to participate in the intervention protocol with veterans; (4) read and speak English; and (5) have access to a telephone.

#### Exclusion criteria

Veterans and caregivers are excluded if they either are < 18 years in age, were not contacted within ten phone calls, deny that they have ADRD or a TBI, have difficulty hearing or speaking by telephone, have a terminal illness, have a history of hospitalization for alcohol or drug abuse or severe mental illness (e.g., suicidal tendencies, severe untreated depression or bipolar disorder, or schizophrenia), or are a prisoner or under house arrest. Caregivers also are excluded if they do not consider themselves an informal caregiver (e.g., believe that the veteran is not impaired or does not support them in any capacity) or if they have a serious medical illness limiting their ability to participate (e.g., suicidal tendencies, severe untreated depression or bipolar disorder, or schizophrenia).

### Recruitment and randomization

We use a computer-generated randomization scheme created by the study statisticians that is stratified by veterans’ primary diagnosis (ADRD versus TBI) to assign dyads rather than providers or clinics to the intervention or control group. Randomization at the dyad level minimizes the effects of unmeasured case mix differences and clinic-level clustering. Based on data from our other randomized studies and the literature, the risk for “spillover” from having participating clinics treat both intervention and usual care patients is likely to be small [27, 33, 34].

### Description of the intervention

ABC ANSWERS has two main goals: (1) comanage and support the practice of primary care providers (PCPs) to care for persons with ADRD or TBI (macro, system-level change) and (2) enhance self-management skills of the caregiver and veteran to maximize their coping behaviors by building on the dyads’ strengths and abilities while compensating for cognitive and functional losses (micro, dyad-level change). The intervention components provide the ability to integrate and connect various ADRD and TBI resources available and are motivated by ADRD care quality standards [35, 36] and supplemented with chronic disease management approaches primarily directed to caregivers. The intervention produces standardized, comprehensive assessments to develop and implement care goals and “set the stage” for VHA PACTs. PACT members are then able to conduct in-depth discussions, primarily with caregivers, to identify symptoms impacting QoL and caregiver burden.

The program has three main phases: an initial assessment phase, a strength-based collaborative care plan development phase, and an ongoing collaborative care management phase. By design, the protocol addresses a wide range of care needs and results in a tailored intervention approach for individual veterans and their caregivers. The program components derive from several core strategies essential for successful care management:

1. Building a relationship and trust with dyads to increase engagement
2. Using a strength-based approach to establish goals that are meaningful and achievable for dyads and the respective care team members
3. Use of brief, structured validated assessments that are comprehensive to increase scalability
4. Increasing “on-demand” caregiver access to program resources
5. Ensuring flexibility in relationships with primary care physicians in establishing allocation of responsibility
6. Ensuring standards of expertise that cover a range of biopsychosocial needs to support the dyads via interdisciplinary care management teams

The program attempts at least one in-person home visit with the dyad and the ABC ANSWERS team during the first 4 weeks after enrollment, supplemented by at least monthly calls for the first 8 weeks and bimonthly calls during the remaining months. Supplemental phone calls can be scheduled with the dyad on an as-needed basis or as directed through the results of the ANSWERS action plan.

#### Initial assessment phase

The ABC ANSWERS team is structured to maximize the skill sets of specially trained nurses and community health workers who function as care managers (CMs) and CM assistants in a collaborative model with the primary care team to maximize effectiveness in improving QoL and decreasing caregiver burden. The team is supervised by a physician with expertise in caring for patients with cognitive disorders. The intervention team conducts a biopsychosocial needs assessment in person or by phone upon enrollment. This assessment includes a demographic and psychosocial interview focused on achieving problem identification. The program uses standardized assessment tools including the Healthy Aging Brain Care Monitor [37]. The team focuses on problem clarification and reviews the assessment findings, medical records, medication lists, and other associated assessments. If the veteran has unspecified cognitive issues as a diagnosis, the CM also reviews diagnostic testing, brain imaging results, and functional details of the assessment to determine the presence or absence of a likely dementia or TBI diagnosis and to identify reversible and comorbid conditions. The team creates an initial plan and identifies areas needing further assessment after the initial home visit. For complex cases, the registered nurse will assess need for referral for further evaluation with mental health, neurology, or geriatrics professionals. Feedback from the initial assessment and home visit are forwarded to the veteran’s PCP with requested feedback and further direction as needed.

#### Collaborative, strength-based care plan development phase

This phase starts after the initial home visit and concludes with the delivery of the nine ANSWERS sessions (Table 1). The goal of this phase is to create an individualized care plan in collaboration with the veteran’s primary care team. After the initial assessment is completed, any urgent medical, behavioral, and/or psychological issues are addressed through consultation with the program ADRD and TBI specialists, as well as with the PCP as needed. During the second visit (in home or by phone), the team reviews the proposed care plan and identified problem list and seeks input from the dyad in prioritizing care issues. From this resulting consensus, the CM discusses the proposed individualized care plan; explains the diagnosis (ADRD or TBI) and natural history; implements appropriate care protocols; reviews, explains, and distributes the corresponding educational handouts for the dyad; and connects veterans and caregivers to eligible services and community resources. Once all urgent needs have been addressed and the care plan is established, the team initiates the first ANSWERS session, shown in Table 1. Each ANSWERS session consists of a 60- to 90-min structured curriculum that provides education, counseling-based skills, and cognitive rehabilitation techniques to veterans and their caregivers for coping with and managing ADRD and TBI. At the end of each session, the ABC ANSWERS team members and the dyad create an action plan, which outlines specific skills and sets of activities that will be practiced until the next session. The action plan states how often a technique should be practiced, defines roles of the veteran and caregiver in practicing each technique, and provides space to document progress and barriers. Sessions 1–6 focus on key topic areas (see Table 1), and sessions 7–9 serve as “booster sessions” that are conducted over the phone to assist with the maintenance, modification, and generalization of skills.

**Table 1:** Aging Brain Care Acquiring New Skills While Enhancing Remaining Strengths intervention content

	Topic	Materials
1	Introduction of project, format of sessions, and goals Overview of educational information about communication, cognitive or thinking abilities, roles, and social activities	Strength-based assessment Skills checklist Review of strength/skills Session evaluation
2	Review of session 1 Introduction to effective communication skills Difficulties in communication Communication skills and techniques	Supplementary session worksheets Examples of skills include patience and acceptance, compromising, KISS, rephrasing questions, redirection with verbal and/or physical cues, narrowing the choices (closed-ended questions), and connecting with others (open-ended questions) Session evaluation Action plan
3	Review and discuss session 2 action plan Introduction to cognitive engagement Discussion tools, tips, and strategies of cognitive engagement	Review session 2 action plan Examples of skills include giving hints, spaced retrieval, and external memory aids (i.e., signs, lists) Supplementary session worksheets Session evaluation Action plan
4	Review and discuss session 3 action plan Introduction to understanding emotions and behaviors	Review session 3 action plan Examples of skills include cognitive task analysis, building on and using previously learned skills, and the activity notebook for cognitive stimulation and engagement Action plan
5	Review and discuss session 4 action plan and reflection Cognitive techniques to manage emotions and behaviors Review of skills from sessions 2 and 3	Review session 4 action plan Skills include validation, reframing, reevaluating expectations, giving yourself permission, substituting behaviors, adjusting the environment, and making time to relax Supplementary session worksheets Session evaluation Action plan
6	Review and discuss sessions 4 and 5 action plans Introduction to managing role changes and social activities Skills and techniques to support roles and activities	Review of action plan Examples of skills include simplifying activities and routines, pleasant activities assessment, increasing pleasant activities Supplementary session worksheets Session evaluation Action plan
7	Review and discuss session 6 action plan Advanced practice for care partners	Review of action plan Session evaluation Action plan
8	Review and discuss session 7 action plan Session wrapup and future action plans	Review of action plan Session evaluation Overall program evaluations Action plan
9	12-week booster session Review and discuss session 8 action plan Session wrapup and future action plans	Review of action plan Session 9 evaluations

#### Follow-up phase

During the follow-up phase, the CM team continues to work with the dyad as outlined by the ANSWERS action plan and based on presenting needs and circumstances. The CM team answers any questions generated from previous visits, collects veteran and caregiver feedback, has the caregiver complete a brief assessment to identify need for specific care protocols, and facilitates caregivers’ participation in an array of community services that are readily available. The CM reconciles medications and reviews medication adherence during both home visits or by phone. Throughout the duration of the follow-up phase, the team continues to work with the dyad and the veteran’s primary care team to monitor, implement, and adjust the individualized care plan as necessary.

### Description of the control

Control group participants do not receive the ABC ANSWERS intervention but receive routine VHA primary care and standardized, written educational and resource information. The written material covers various topics related to symptoms of ADRD and TBI, treatments, managing the disease at home, caregiving, and VHA community agencies that can be independently contacted for assistance [38].

### Primary outcome measures

Caregiver QoL is the primary outcome measure. Secondary outcome measures include caregiver symptoms of depression, anxiety, and caregiver burden. Additional secondary measures for the trial include veteran QoL and symptoms of depression and anxiety. In addition to the psychosocial outcomes data, several additional types of data addressing the implementation and translation process also are examined. Three follow-up data collection periods occur following the baseline (0 months) data collection: short-term (3 months), intermediate (6 months), and sustained (12 months) periods.

#### QoL

The primary outcome is QoL (Quality of Life in Alzheimer’s Disease scale [QOL-AD]) measured with a 13-item scale [39, 40] designed to provide both a patient report and a caregiver report of QoL [39, 40]. Responses are structured in a four-choice format (i.e., poor, fair, good, or excellent) that is consistent across all questions, and all items are rated according to the caregiver’s or veteran’s current QoL. The scale includes appraisal of physical QoL, mood, interpersonal relationships, ability to participate in meaningful activities, financial situation, and overall assessment of QoL. Psychometric properties indicated that the QOL-AD has both content validity and criterion content validity when correlated with the Dementia Quality of Life Instrument (*r* = 0.69) and the EuroQol EQ-5D scale (*r* = 0.54). While QOL-AD has good construct validity, both interrater reliability (Cohen’s kappa > 0.70) and internal consistency (Cronbach’s alpha coefficient = 0.82) were excellent [41]. In a more recent evaluation, the QOL-AD demonstrated acceptable reproducibility at 2 weeks with intraclass correlation coefficient (ICC) > 0.80 and internal consistency with Cronbach’s alpha coefficient > 0.70. In fact, Wolak-Thierry et al. [42] reported that the QOL-AD was preferred over the general health dimension of the Duke Health Profile because the QOL-AD was significantly (*P* < 0.0001) more efficient in time required to complete [41, 42].

#### Caregiver burden

The Oberst Caregiving Burden Scale (OCBS) [43] is used to measure (1) caregiving tasks and responsibilities, (2) relationships and interpersonal support, (3) lifestyle, (4) emotional and physical health, and (5) overall personal impact of being a caregiver. OCBS is a reliable and valid questionnaire that rates 15 different caregiving tasks of informal caregivers based on perceived time and difficulty of the task (e.g., providing personal and medical care, assisting with ADRDLs, monitoring symptoms, managing patient’s emotions and behaviors, dealing with finances, and coordinating and seeking health services). Each item is scored on a 5-point response scale. In response to time for each task, the caregiver chooses an amount ranging from no time to a great deal of time spent on the task related to caregiving. In response to difficulty for each task, the caregiver chooses a value ranging from not difficult to extremely difficult. Similarly, there is a subscale regarding level of distress associated with each task.

Subscale scores for perceived time, difficulty, and distress will be obtained by summing across the 15 items scored 1 to 5. Thus, subscale scores will have a range of 15–75, with higher values indicating more perceived time, difficulty, and distress with caregiving tasks. Evidence of reliability, content validity, and construct validity for the OCBS were reported in prior studies of ADRD [44–46] and TBI [47] family caregivers and people caring for persons receiving cancer treatment [48–50]. Acceptable internal consistency reliability also has been reported in the context of family caregivers of stroke survivors [51, 52].

#### Depressive and anxiety symptoms

We use the 10-item Center for Epidemiologic Studies Depression Scale (CES-D) [53–55] to determine the impact of the ABC ANSWERS intervention on caregivers’ and veterans’ depression. The CES-D is a 10-item depression scale with a total score ranging from 0 to 30, with higher scores indicating more symptoms of depression and a score ≥ 10 indicating depression [55]. To measure anxiety symptoms, we use the Generalized Anxiety Disorder 7-item scale (GAD-7), which is a 7-item anxiety scale with a total score ranging from 0 to 21 [56]. Both the CES-D and the GAD-7 have good internal consistency and test–retest reliability, as well as convergent, construct, criterion, procedural, and factorial validity for the diagnosis of major depression and general anxiety disorder [56–58]. We have used both instruments in multiple research studies, including our dementia collaborative care trials [2, 4, 10, 25–28, 30, 31, 56, 57].

#### Other measures

In addition to the primary and secondary measures described above, we also will measure caregiver and veteran satisfaction with life [59–62]; caregiver’s emotional and physical health strain, feelings of dyadic strain, and role captivity; and the amount of veteran’s distress experienced due to difficulties experienced in completing ADL [63–66].

### Data collection

Research assistants collect survey data by phone or in person at the VHA and are blinded to which condition participants are assigned. Caregivers and veterans are interviewed separately, and each assessment lasts approximately 35–45 min. Research assistants use protocols followed in prior studies to facilitate veterans’ comprehension and participation [67]. Once the assessments are completed, veterans and their caregivers receive $20 gift cards to compensate them for their time in participating in each study interview. To promote participant retention, research assistants call both members of the study dyad to schedule a convenient time to complete outcome assessments. The research team will attempt to contact participants ten times during the 4-week outcome assessment time frame. If a dyad withdraws from the study before their 12-month duration is over, they will no longer be pursued for outcome data. Any outcome data gathered from the dyad while they were enrolled and active in the study will be used for the analyses.

Research assistants attend a 1-day comprehensive training session lead by the principal investigator (PI) and complete two practice interviews with the PI or a coinvestigator who is observing. Training includes all procedures, questionnaires, and the written manual as a reference guide. Topics covered during the training include human subject protection, secure data management, VHA suicide protocol and informed consent, Health Insurance Portability and Accountability Act (HIPAA; Public Law 104–191) guidelines, confidentiality of responses, techniques for interviewing older adults and individuals with ADRD and TBI, and protocols for interviewer assignments and returning completed interviews. All data assessments are reviewed and verified by a second team member before being finalized in the secure RedCap database that sits behind the VHA firewall. Data safety and monitoring will occur at the monthly investigators group meeting. This group will monitor recruitment, enrollment, data collection, intervention procedures, and participant safety issues such as adverse events and protocol deviations on an ad hoc basis. This is a minimal risk study, and we do not anticipate any study-related serious adverse events. However, we will track all adverse events and will report any serious adverse events to the IRB as a reportable VHA event per VHA policy. At continuing review, the IRB will receive a copy of all adverse events not requiring prompt reporting. PHI will not be shared during data safety and monitoring discussions.

### Analysis plan

Randomization results will be compared with a preplanned randomization schedule to ensure randomization integrity. To verify comparability of the randomized groups, baseline characteristics (age, sex, race, and education level) will be compared between randomized groups for veterans and caregivers separately as well as veterans’ comorbid medical conditions, the Charlson comorbidity index, number of primary care visits, and acute care use during the year prior to enrollment. This analysis will be conducted using analysis of covariance models for continuous variables and the Cochran-Mantel-Hansel statistic for categorical variables while controlling for the stratification variable of primary diagnosis (ADRD or TBI). The distributions of continuous variables will be examined, and transformation or nonparametric methods will be used in cases of violation to the normal distribution assumption. In addition, the frequency distribution will be examined for all categorical variables, and exact inference procedures will be used in cases of zero or small cell size. All analyses will be conducted with SAS 9.4 software (SAS Institute, Cary, NC, USA).

Mixed effects models will be used with longitudinally collected QoL scores from veterans as the dependent variable, and randomization group, time, and interaction between group and time as the independent variables while adjusting for the stratification variable of primary diagnosis (ADRD or TBI) and baseline variables that are found to be different between the two groups. An unstructured variance–covariance matrix will be used in the mixed effects model to adjust for potential correlations among measures obtained from the same individual over time. A significant interaction between group and time would indicate differences in QoL changes over time between the ABC ANSWER and control groups. Linear contrasts will be used to compare the QoL scores at each follow-up time between the two groups. Absent significant interaction, significant main group effects would suggest differences in QoL measures between the two groups across all follow-up times. Parametric estimation and inference procedures for the mixed effects models are conducted using the maximum likelihood approach with robust parametric estimation and inference under many missing data mechanisms [68]. In addition, other covariates will be included in the mixed effects models to determine whether specific family member characteristics (e.g., relationship to veteran, frequency or types of contact, other people living in the household) are associated with QoL changes over time. Similar analysis will be conducted for the outcome of caregiver QoL.

Additionally, a mixed effects model will be conducted with caregiver QoL as the outcome variable and veterans’ QoL measures as an additional independent variable to determine how much of the intervention effect on caregiver QoL is mediated through changes in veterans’ QoL measures. By realigning veteran QoL and caregiver QoL, we can also determine whether any mediation effect from veteran QoL is concurrent or has lagged in time.

Similar linear mixed models will be fit for outcomes of caregiver burden (OCBS) and outcomes collected on both veteran and caregiver: depression (CES-D), anxiety (GAD-7), and dyadic strain (dyadic relationship strain and role captivity). All veteran and caregiver outcomes will be fit with separate models. Linear contrasts obtained from the models will be the differences in mean score between the intervention group and the control group at each follow-up time.

For dyad analyses, we will use the dyadic nature of the study design and assess how caregivers’ outcomes are related to veterans’ measures. We will first explore the degree of concordance between paired veteran–caregiver reports using mixed effects models with concurrent veterans’ measures from the same time points as measures of caregivers. ICCs between veteran and caregiver measures will be calculated to assess the degree of chance-corrected agreement between individual veteran and caregiver outcomes, controlling for within-person correlations over time. Mixed effects models will also be used to explore potential mediation effect of the intervention on caregivers’ outcomes from the effect on veterans’ outcomes. For example, we will examine whether intervention effect on caregiver QoL, burden score, and depressive or anxiety symptoms is mediated by an increase in veterans’ QoL or decreased depressive or anxiety symptoms in the intervention group versus the control group. No interim analyses are planned.

#### Statistical power

Power estimation was conducted using the GLMPOWER procedure in SAS 9.4 software, which takes into account the repeated measures. Power was estimated under two scenarios for potential intervention effects for the primary outcome of veteran QoL. In the first scenario, we assume an early intervention effect with a 0.3 standard deviation (SD) effect size at 3 months and sustained intervention effects of 0.3 SD at 6 and 12 months. In the second scenario, we assume that the effect of intervention is gradual in a dose–response fashion (i.e., 0.17 SD, 0.34 SD, and 0.68 SD effect sizes at 3, 6, and 12 months, respectively). We further assumed a Toeplitz pattern for the correlation structure with correlations of 0.4 for adjacent measures and 0.2 for measures farther apart. With the total enrollment of 200 veteran–caregiver dyads, we expect 10% loss to follow-up rate due to refusal or death. With 180 veteran–caregiver dyads remaining, both scenarios will have > 80% power to detect an intervention effect from a linear mixed model with type I error set at 0.05. Power will be similar for other aims.

### Dissemination plan

A detailed study protocol will be published in an open-access journal. Any modifications made to the protocol will be communicated to Indiana University, Veteran Affairs Medical Center Indianapolis VA Research Subcommittee, and U.S. Army Medical Research and Material Command, Office of Research Protections. The findings of this study will also be presented at scientific conferences. Also, a manuscript will be prepared and submitted to a peer-reviewed scientific journal for possible publication.

## Discussion

Over the past 20 years, interventions aimed at improving QoL for caregivers and patients and reducing caregiver burden have moved progressively, but separately, from testing educational and skills training to cognitive training for persons with cognitive impairment and to complex collaborative care models [2, 4, 10, 25–28, 30–32, 56, 57]. This progression has created both micro (patient–caregiver dyad-focused) and macro (population health–focused) interventions that have brought a greater number of resources into primary care. Unfortunately, research also has revealed practical constraints of time and space and lack of expertise in caring for people with ADRD and TBI within the primary care environment. Thus, new models of care are moving toward a view of primary care as the “hub of care” (e.g., PACTs), which is the cornerstone of the new models of care transformation in VHA [2, 69, 70]. One implication of this paradigm shift is that models of care for people with ADRD and TBI must be expanded to include key components of population brain health that are implemented collaboratively in primary care. However, these models have not been widely adopted, because they require a redesign of the practice environment and partnerships with the health care system that support practice redesign.

VHA PACTs provide the ideal environment to test these new models of collaborative care for ADRD and TBI. Thus, we have designed ABC ANSWERS to assist the PACTs in achieving recommended standards of care through evaluation and management of patients with ADRD and TBI and to support and improve the coping, well-being, and independence of veterans with ADRD and TBI and their caregivers.

The ABC ANSWERS intervention integrates the common features of an evidence-based collaborative care model for brain care while also attending to the implementation barriers of delivering care and skills to dyads of veterans with ADRD or TBI and their caregivers. To date, our work has resulted in significant improvement in the quality of care and management of behavioral and psychological symptoms for patients and their caregivers, and it has significantly reduced caregivers’ strain and improved caregivers’ well-being and their sense of mastery in a caregiver role [2, 4, 10, 25–28, 30–32, 56, 57].

The ABC ANSWERS trial has some limitations. The most significant threat may be lack of ability to recruit because of the underdetection of ADRD and TBI among veterans. This would limit our ability to recruit dyads in which a veteran has one of these conditions and could be enrolled [71, 72]. To address this potential limitation, we have opened recruitment from all primary care sources and include veterans who have been seen at VHA specialty clinics and programs as long as they also receive primary care from a VHA provider. A second possible limitation of our study is the variable interactions between the ABC ANSWERS care team and the various primary care PACTs. Findings may reflect different culture among the PACTs and personal qualities and relationship of the team’s interactions. However, a strong protocol-driven intervention and standardized training program will limit quality and reproducibility concerns. Furthermore, dyads randomized to the usual primary care group may receive nonstudy ADRD and TBI resources from their PACTs or resources from other VHA services. However, it is unlikely that dyads will receive management strategies that reflect the constructs in the ABC ANSWERS intervention through their engagement with the PACTs for usual care.

In summary, the tremendous need for primary care–based interventions that are focused on dyads and aim to improve care and outcomes for both people with ADRD and TBI and their caregivers remains. ABC ANSWERS aims to test the effectiveness of the intervention for improving QoL and reducing burden through care coordination, psychosocial well-being outcomes, and reducing different types of role and intrapsychic strain. The results of this study will inform other interventions that support patients with ADRD or TBI and their caregivers and that are scalable and embedded in existing VA primary care.

## Supplementary information

**Additional file 1.** SPIRIT 2013 checklist: recommended items to address in a clinical trial protocol and related documents.

## Data Availability

Any data required to support the protocol can be supplied upon request.

## Abbreviations

ABC ANSWERS: Aging Brain Care Acquiring New Skills While Enhancing Remaining Strengths
ADLs: Activities of daily living
ADRD: Alzheimer’s disease and related dementias
AMA-PCPI: American Medical Association Physician Consortium for Quality Practice
CES-D: Center for Epidemiologic Studies Depression Scale
CM: Care manager
DMWG: Dementia Measures Work Group
GAD-7: Generalized Anxiety Disorder 7-item scale
ICC: Intraclass correlation coefficient
IRB: Institutional review board
OCBS: Oberst Caregiving Burden Scale
PACT: Patient Aligned Care Team
PCP: Primary care provider
PI: Principal investigator
QoL: Quality of life
QOL-AD: Quality of Life in Alzheimer’s Disease scale
TBI: Traumatic brain injury
VHA: Veterans Health Administration
